# MMPred: a tool to predict peptide mimicry events in MHC class II recognition

**DOI:** 10.3389/fgene.2024.1500684

**Published:** 2024-12-10

**Authors:** Filippo Guerri, Valentin Junet, Judith Farrés, Xavier Daura

**Affiliations:** ^1^ Anaxomics Biotech, Barcelona, Spain; ^2^ Institute of Biotechnology and Biomedicine, Universitat Autònoma de Barcelona, Cerdanyola del Vallès, Spain; ^3^ Catalan Institution for Research and Advanced Studies (ICREA), Barcelona, Spain; ^4^ Centro de Investigación Biomédica en Red de Bioingeniería, Biomateriales y Nanomedicina, Instituto de Salud Carlos III, Cerdanyola del Vallès, Spain

**Keywords:** molecular mimicry, epitope prediction, sequence alignment, MHC class II, autoimmune disease, SARS-CoV-2

## Abstract

We present MMPred, a software tool that integrates epitope prediction and sequence alignment algorithms to streamline the computational analysis of molecular mimicry events in autoimmune diseases. Starting with two protein or peptide sets (e.g., from human and SARS-CoV-2), MMPred facilitates the generation, investigation, and testing of mimicry hypotheses by providing epitope predictions specifically for MHC class II alleles, which are frequently implicated in autoimmunity. However, the tool is easily extendable to MHC class I predictions by incorporating pre-trained models from CNN-PepPred and NetMHCpan. To evaluate MMPred’s ability to produce biologically meaningful insights, we conducted a comprehensive assessment involving *i*) predicting associations between known HLA class II human autoepitopes and microbial-peptide mimicry, *ii*) interpreting these predictions within a systems biology framework to identify potential functional links between the predicted autoantigens and pathophysiological pathways related to autoimmune diseases, and *iii*) analyzing illustrative cases in the context of SARS-CoV-2 infection and autoimmunity. MMPred code and user guide are made freely available at https://github.com/ComputBiol-IBB/MMPRED.

## 1 Introduction

Epidemiological, clinical, and experimental evidence supports the association between infections and autoimmune diseases, with molecular mimicry proposed as one of the key mechanisms underlying this relationship (Oldstone, 1998; Rojas et al., 2018). Molecular mimicry occurs when a pathogen-derived peptide shares sequence similarity with a host peptide, and it is considered a strategy employed by pathogens to evade the immune system. By mimicking self-protein sequence fragments, pathogens exploit the immune system’s tolerance for these molecules, effectively avoiding detection. This mimicry can mislead the immune response, activating autoreactive T-cells and/or producing cross-reactive antibodies that target both the pathogen’s peptides and the host’s own tissues. As a result, the immune system may inadvertently attack the body, contributing to the onset of autoimmune disorders. This mechanism is known to play a role in diseases such as Guillain-Barré syndrome (Maguire et al., 2024) or rheumatic fever (Cunningham, 2014), where infections by specific viruses or bacteria, respectively, are linked to autoimmunity. The identification of molecular mimicry events is challenging due to the vast number of potential pathogen-derived peptides and the limited number of known autoantigens.

The identification or prediction of peptide mimicry events could therefore serve as a valuable clinical tool. From a theoretical standpoint, predicting peptide mimicry involves identifying similarities between self and exogenous proteins (Doxey and McConkey, 2013) and predicting their recognition by the immune system (Rojas et al., 2018). In this context, bioinformatics can provide valuable insights through the application of sequence alignment and epitope-prediction algorithms, particularly for Major Histocompatibility Complex (MHC) epitopes (Caron et al., 2015; Wang et al., 2010). To our knowledge, CRESSP (An et al., 2022) is the only tool currently integrating and streamlining both sequence alignment and epitope prediction. However, CRESSP primarily focuses on B-cell epitopes. Although the SARS-CoV-2 study by An et al. (2022) included MHC class II epitopes as well, the actual tool (https://pypi.org/project/cressp/) only incorporates MHC class I epitope predictions, as provided by MHCflurry (O’Donnell et al., 2020).

Here, we present the program MMPred (Molecular Mimicry Predictor), a tool that integrates sequence alignment and MHC class II epitope-prediction algorithms into a single pipeline. The tool is designed to be flexible, user-friendly and amenable to non-expert use, requiring as sole inputs the fasta files for the exogenous (query) and endogenous (target) protein (or peptide) sets and a list of MHC alleles of interest to perform the predictions for. The application of sequence alignment is optional, and if used, the two sets of sequences are compared and epitope prediction is applied to those target sequences that show a significant alignment with query sequences. The tool is an extension of CNN-PepPred (Junet and Daura, 2021), and offers the possibility to include predictions from NetMHCIIpan4.1, both with the BA (trained on binding affinities) and EL (trained on eluted ligands) models (Reynisson et al., 2020). The tool is programmed in such a way that additional predictors, including MHC class I epitope predictors, can be added with minimal effort. The alignments can be performed with BLASTp (Altschul et al., 1990) or by means of a position-specific scoring matrix (PSSM) with PSI-BLAST (Schäffer et al., 2001).

We have evaluated the capacity of MMPred to produce biologically meaningful results by *i*) predicting the association of known HLA class II human autoepitopes to microbial-peptide mimicry, *ii*) evaluating and interpreting these predictions in a systems biology framework, where the predicted autoantigens were tested for possible functional relations to pathopysiological pathways associated to autoimmune disease, and *iii*) analyzing example cases in the context of autoimmunity and SARS-CoV-2 infection (Ehrenfeld et al., 2020; Knight et al., 2021). We note that a statistical evaluation of the performance of the tool would not be meaningful in this case since *i*) the number of human peptides that are know to be (and not be) autoreactive as a result of microbial-peptide mimicry is very small and *ii*) we would be mostly evaluating the prediction performance of CNN-PepPred and NetMHCIIpan, which has been already done and is not the purpose of this study. For the alignment strategy, both BLASTp and PSI-BLAST produced biologically relevant matches. Although PSI-BLAST yielded higher average scores in the functional evaluation, it significantly reduced the number of hits at the same significance threshold. Therefore, we recommend using both alignment methods for a more comprehensive analysis. We discuss some of the predicted autoantigens in further detail, with helicase MOV-10 standing as one of the most interesting cases.

## 2 Methods

### 2.1 MMPred

This study introduces MMPred, a software tool integrating epitope prediction and sequence alignment algorithms to simplify the setup of computational analyses aimed at the generation, investigation or testing of hypotheses relative to molecular mimicry events in the context of autoimmune diseases. As it stands, the tool provides epitope predictions for MHC class II only, as alleles involved in autoimmunity belong often to this class (Fiorillo et al., 2017). But the tool can be easily extended to MHC Class I prediction by incorporating the corresponding pre-trained models from CNN-PepPred and NetMHCpan (Reynisson et al., 2020).

### 2.2 Algorithm

**FIGURE 1:**
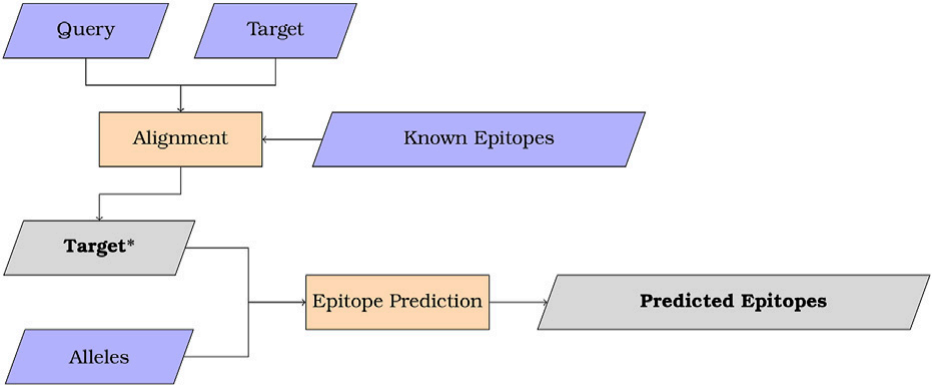
Scheme summarizing the overall workflow of MMPred when used with alignment. Input in purple, process in orange and output in grey.

The MMPred algorithm is illustrated in Figure 1 for uses combining sequence alignment and epitope prediction. In addition, the program may be also used without the alignment feature to streamline epitope prediction using NetMHCIIpan and CNN-PepPred.

#### 2.2.1 Software specifications

The software has been developed using Python3.6.8 in a Linux environment. The Python3 libraries used are Pandas (https://pandas.pydata.org/), NumPy (Harris et al., 2020), Matplotlib (Hunter, 2007), pickle (https://github.com/python/cpython/blob/3.6/Lib/pickle.py), sklearn (Pedregosa et al., 2011) and tensorflow (Abadi et al., 2016). Software, installation instructions and program user-guide are available in GitHub (https://github.com/ComputBiol-IBB/MMPRED).

#### 2.2.2 Input

The program takes a set of protein sequences in the form of a fasta file (QUERY) and a list of MHC alleles (ALLELES) as minimal input. If no additional input is provided, the program runs an epitope prediction for the sequences in QUERY and each of the MHC alleles specified.

The user can specify if QUERY contains entire protein sequences (*protein* mode) or peptides (*peptide* mode). When in *protein* mode, the program makes a prediction for each fragment of size W in the protein (with a 1-residue step), where W is a user-defined parameter. Instead, when using the *peptide* mode a single prediction is performed for the full length of the peptide. We suggest to use the *peptide* mode only when QUERY contains short-length peptides, e.g., ≤ 25 residues.

The user can provide a second fasta file (TARGET). In this case, an alignment is performed between the peptides in QUERY (if in *peptide* mode) or derived from QUERY (if in *protein* mode) and the TARGET sequences, to produce the TARGET* set (see Figure 1). TARGET* contains the sequences from TARGET that show significant (to a value defined by the user) alignment with QUERY peptides. Epitope prediction is then applied to the TARGET* sequences.

In this study, we are using microbial sequences as QUERY and human ones as TARGET. Yet, QUERY and TARGET may be whatever the user thinks appropriate for the analysis in question.

#### 2.2.3 Alignment

Protein sequence alignment is performed to identify the potential similarity between QUERY and TARGET sequences, if TARGET is specified. The alignment can be done using either of two strategies: *i*) using BLASTp to perform an ungapped alignment, with automatic adjustment of parameters for short input sequences or *ii*) using PSI-BLAST to compute a PSSM by aligning the sequences in QUERY against a user-defined set of epitopes provided in a fasta file, and then using the PSSM to perform a search in TARGET. This user-defined epitope set is identified in Figure 1 as *Known Epitopes*, and an example set used in this study is described in Section 2.3.5.

If the alignment satisfies a certain significance threshold (either E-value or bit score; by default E-Value < 0.05) the aligned TARGET sequence is stored in TARGET* for epitope prediction. The parameter (E-value or bit score) and its value can be defined by the user.

To perform the epitope prediction, a sequence of length ≥W that we shall call “prediction window” has to be extracted from the alignment. If the length of the alignment is <W , then a prediction window of size W centered on the aligned sequence is extracted. If the number of extra residues at left and right cannot be the same, the program automatically takes the extra residue to the right. If the window falls outside the ends of the sequence, the algorithm will take the first or last W residues accordingly. W is by default 15.

#### 2.2.4 Epitope prediction

Whether the alignment is performed or not, epitope predictions are run using NetMHCIIpan 4.1, with both the model trained on Binding Affinity (BA) and that trained on mass spectrometry Eluted Ligands (EL) (Reynisson et al., 2020), and CNN-PepPred (Junet and Daura, 2021). The three predictions are kept and no consensus score is generated. Note that NetMHCIIpan reports the prediction score and the %Rank for each peptide-MHC pair. The %Rank is a normalized prediction score that enables comparison between different MHC alleles and models (BA and EL). The %Rank of a query sequence is determined by comparing the prediction score to a score distribution for a random set of natural peptides, with %Rank = 1 meaning that the queried sequence obtained a prediction score in the highest 1% of the distribution. On the other hand, CNN-PepPred reports only prediction scores. To make results from CNN-PepPred comparable to NetMhcIIpan, a score distribution for natural peptides was generated for each MHC allele available in CNN-PepPred, using a random sample of 10,000 peptides extracted from UniRef50 (The UniProt Consortium, 2023).

#### 2.2.5 Output

The output depends on the input parameters as follows:

• *No Alignment, Protein mode:* The program will return the predicted binding core (a sequence of length 9 for MHC class II binders (Wu et al., 2021)) for every window of size W achieving %Rank ≤ 10 in every protein included in QUERY, together with the ID of the protein, start and end position of the W -residue peptide, start and end position of the predicted core, prediction method, score, %Rank and the allele for which the prediction has been made.

• *No Alignment, Peptide mode:* The program will return the predicted 9-mer core for every peptide included in QUERY achieving %Rank ≤ 10, together with the ID of the peptide, start and end position of the predicted core, prediction method, score, %Rank and the allele for which the prediction has been made.

• *Alignment*: For every sequence in TARGET* achieving %Rank ≤ 10, the program will return the predicted 9-mer core, the TARGET sequence ID, the start and end position of the alignment in the TARGET sequence, the start and end position of the window of size W extracted from the alignment in the TARGET sequence, the start and end position of the predicted core in the TARGET sequence, identity, E-value and bit score of the alignment, the aligned TARGET sequence, the QUERY sequence ID, the start and end position in the QUERY sequence of the alignment, the aligned QUERY sequence, prediction method, score, %Rank and the allele for which the prediction has been made.

### 2.3 Evaluation datasets

#### 2.3.1 Microbial epitope dataset (MEDS)

A dataset of known HLA class II epitopes from infectious agents was manually downloaded from IEDB (Vita et al., 2019) (https://www.iedb.org). The search terms “Epitope: Linear peptide”, “Epitope source: Bacteria, Virus”, “Host: Human”, “Assay: MHC ligand”, “Outcome: positive”, “MHC Restriction: Class II”, “Disease: any” were used (date 11/10/2023).

Epitopes lacking either the UniProtKB accession number (AC) (The UniProt Consortium, 2023) (https://www.uniprot.org) of the protein, start or end position in the sequence or the HLA allele for which they were tested, were excluded. Epitopes containing modified amino-acid residues were also discarded. Additionally, only epitopes from microorganisms known to be related to autoimmune diseases were considered. The relationship between an organism and the occurrence of autoimmune diseases was determined by review of the literature when not annotated in IEDB. For each organism with epitopes fulfilling the previous selection criteria, a PubMed (https://pubmed.ncbi.nlm.nih.gov/) search for reviews from the last 10 years using the keywords “organism_name AND (autoimmunity OR autoimmune)” was performed. As last filter, epitopes associated to HLA alleles with no model available in CNN-PepPred and NetMHCIIPan were also discarded.

After these filters, MEDS contained 3,676 epitopes from 88 proteins of 13 microorganisms, associated to 50 HLA class II alleles, with a total of 9,229 epitope-allele pairs (Supplementary Material Table 1 file MEDS_summary.xslx).

#### 2.3.2 Human autoepitope DataSet (HADS)

A dataset containing known human autoepitopes was downloaded from IEDB (11/10/2023). The search terms were: “Epitope: Linear peptide”, “Epitope source: Human”, “Host: Human”, “Assay: MHC ligand”, “Outcome: positive”, “MHC Restriction: Class II”, “Disease: autoimmune”.

To avoid redundancy in the form of nested sets, epitopes from the same protein that showed overlap and were linked to the same allele were merged into a single epitope. After the merging, only sequences with length ≥ 15 were kept. Epitopes associated to HLA alleles with no model available in CNN-PepPred and NetMHCIIPan were also discarded.

HADS thus contained 807 epitopes from 608 different human proteins, associated to 5 different HLA class II alleles in the context of Rheumatoyd Arthritis and Multiple Sclerosis (Supplementary Material Table 2 file HADS_summary.xslx).

#### 2.3.3 Human proteome dataset (HPDS)

HPDS contains the sequences of the 20,426 reviewed human proteins found in UniProt (The UniProt Consortium, 2023) on the date of the download (18/10/2023).

#### 2.3.4 SARS-CoV-2 proteome dataset (SC2DS)

SC2DS was generated from the SARS-CoV-2 reference proteome (Wu et al., 2020) (UniProt identifier: UP000464024, downloaded 11/01/2024) and the Pangolin variants (Rambaut et al., 2020) (downloaded 01/03/2024) A.23.1-like, A.23.1-like + E484K, Alpha_(B.1.1.7-like), AV.1-like, B.1.1.318-like, B.1.1.7-like + E484K, B.1.617.1-like, B.1.617.3-like, Beta_(B.1.351-like), Delta_(AY.4.2-like), Delta_(AY.4-like), Delta_(B.1.617.2-like), Delta_(B.1.617.2-like)_+K417N, Epsilon_(B.1.427-like), Epsilon_(B.1.429-like), Eta_(B.1.525-like), Gamma_(P.1-like), Iota_(B.1.526-like), Lambda_(C.37-like), Mu_(B.1.621-like), Omicron_(BA.1-like), Omicron_(BA.2-like), Omicron_(BA.3-like), Omicron_(BA.4-like), Omicron_(BA.5-like), Omicron_(Unassigned), Omicron_(XBB.1.16-like), Omicron_(XBB.1.5-like), Omicron_(XBB.1-like), Omicron_(XBB-like), Theta_(P.3-like), XBB-parent1, XBB-parent2, XE-parent1, XE-parent2, Zeta_(P.2-like). The downloaded sequences were manually split into all possible overlapping fragments of size 15. Fragments of variant sequences were only kept if they had indels or mutations relative to the reference proteome. SC2DS thus contained 9,608 15-mers, of which 243 from Pangolin variants.

#### 2.3.5 MHC class II epitopes dataset (MHCII-EDS)

MHCII-EDS contains all MHC class II epitopes available in IEDB (date 18/03/2024), obtained with the search terms: “Epitope: Linear peptide”, “Epitope source: Any”, “Host: Any”, “Assay: MHC ligand”, “Outcome: positive”, “MHC Restriction: Class II”, “Disease: Any”. A total of 485,020 epitopes were downloaded. Redundancy was eliminated by clustering the sequences at 95% identity with CD-HIT (Li and Godzik, 2006), then using the centroid as cluster representative to obtain a final set of 155,923 epitopes. This dataset was used for the computation of the PSSM in all the analyses where PSI-BLAST was used as alignment algorithm.

### 2.4 MMPred evaluation

#### 2.4.1 Evaluation setup

To evaluate the algorithm, five sets of predictions were obtained:

1. Sequences from the human autoepitope dataset (HADS) (TARGET) that significantly align with sequences from the microbial epitope dataset (MEDS) (QUERY) using BLASTp and were positive for binding to HLA class II (with or without allele match, see below) with CNN-PepPred and/or NetMHCIIpan.
2. Same as prediction set 1 but using PSI-BLAST for the alignment.
3. Same as prediction set 1 but with the human proteome dataset (HPDS) as TARGET.
4. Same as prediction set 3 but using PSI-BLAST for the alignment.
5. Sequences from HPDS (TARGET) that significantly align with sequences from the SARS-CoV-2 proteome dataset (SC2DS) (QUERY) using BLASTp and where positive for binding to HLA class II with CNN-PepPred and/or NetMHCIIpan.
6. Same as prediction set 5 but using PSI-BLAST for the alignment.

Prediction sets 1 and 2 were based on a threshold E-value of 0.05 for the alignments and a %Rank ≤ 2 as condition for binding—Reynisson et al. (2020) defined peptides with %Rank ≤ 2 as strong binders and peptides with 2 < %Rank ≤ 10 as weak binders. Prediction sets 3 and 4 were instead obtained in replicates by using threshold E-values from 0.1 to 0.001 and threshold %Rank values of 2 and 10. Owing to the results obtained for prediction sets 3 and 4, prediction sets 5 and 6 were based on a threshold E-value of 0.01 for the alignments and a %Rank ≤ 2.

Prediction sets 1 to 4 were evaluated according to two allele-selection criteria:

1. AllHLA: epitope prediction for HADS (or HPDS) sequences that had at least one significant alignment with MEDS sequences was performed for all alleles.
2. OneHLA: epitope prediction for HADS (or HPDS) sequences that had at least one significant alignment with MEDS sequences was only performed for the allele(s) corresponding to the microbial epitope-allele pair(s) indicated in MEDS.

Furthermore, they were also evaluated both ignoring and considering allele matches:

1. *Epitope* prediction: sequences in HADS (or HPDS) were considered to be predicted as mimicry-induced autoepitopes if there was at least one alignment with MEDS sequences that satisfied the threshold E-value and there was at least one prediction from CNN-PepPred or NetMHCIIpan (BA or EL models) that satisfied the threshold %Rank for any HLA allele.
2. *Epitope-allele* prediction: epitope-allele pairs in HADS (or HPDS) were considered to be predicted as autoepitope-allele pairs if there was at least one alignment with MEDS that satisfied the threshold E-value, and there was at least one prediction from CNN-PepPred or NetMHCIIpan (BA or EL models) that satisfied the threshold %Rank for the same HLA allele of the MEDS pair.

#### 2.4.2 Prediction sets 1 and 2: Supervised evaluation

Prediction sets 1 and 2 may be viewed as a supervised evaluation, since the TARGET sequences are labeled, i.e., they are known to be human autoepitopes, albeit not necessarily relatable to infection events. The remaining prediction sets have the entire, unlabeled human proteome (HPDS) as TARGET.

The allele set used for prediction contained all alleles for which a model exists in either CNN-PepPred or NetMHCIIpan and are present in either HADS or MEDS, totalling 58 alleles (see Supplementary Material Table 3 file Alleles.xslx).

#### 2.4.3 Prediction sets 3 and 4: functional evaluation

Prediction sets 3 and 4 were used to investigate potential functional relationships between predicted autoantigens and the pathophysiological pathways associated with specific autoimmune diseases. This investigation involved a post-analysis of the predicted autoantigens using a systems biology approach. This approach, detailed in Segú-Vergés et al. (2022), evaluates the likelihood of a functional relationship between a given protein and a set of proteins based on their connections within a model of the human protein network. The underlying algorithm employs a supervised machine-learning technique, specifically Artificial Neural Networks (ANNs), trained on a large dataset comprising pharmacological drug targets and molecular descriptors of clinical phenotypes—such as drug indications and adverse effects–from the Biological Effector Database (BED (Jorba et al., 2020)) compiled by the authors.

In BED, each condition (e.g., giant-cell arteritis) is characterized by a list of motifs (e.g., dysfunction of immune checkpoints), and each motif is linked to a set of proteins that map to a specific subgraph within the network. The training objective of the ANNs is to correctly associate drug targets with their respective clinical conditions. The resulting score, S , represents the probability that a given relationship is a true positive, expressed as a percentage. Here, a “relationship” refers to a scenario where a perturbation of one element (e.g., a drug target) leads to an observable perturbation in another (e.g., a clinical condition).

Although the algorithm was originally trained on drug targets rather than autoantigens, it is designed to assign probabilities to the association of any node within a protein network with a specific subgraph. Since the propagation of perturbations or signals between proteins operates on the same principles regardless of whether the context involves drug targets or other protein types, the algorithm can calculate the probability of a relationship between any protein in the network, such as a potential autoantigen, and any clinical conditions annotated in the network, such as autoimmune diseases.

Two protein-network topologies are available for the ANNs. In one of them, the functional associations are based on experimental evidence; in the other, they are based on inference and computed using a variety of resources (protein-protein interactions, gene expression, etc.). The analysis is run for both topologies and the largest score is taken. The score S for a given protein-condition/motif pair may be zero when the protein is not present in the network or no possible association is found for the pair. In such case, the pair is removed from the analysis.

In this study, S was determined for the relationship between each of the human proteins from HPDS that we predicted to be autoantigens as a consequence of microbial peptide mimicry and a list of patophysiological motifs characteristic of each selected autoimmune disease. The list of autoimmune diseases was compiled from the same articles used for the generation of MEDS (see Section 2.3.1), and each autoimmune disease was then mapped to corresponding pathophysiological motifs compiled in BED (see MEDS_summary.xlsx in Supplementary Material Table 1).

For a given BED condition, both the separate protein sets corresponding to the individual motifs and a single protein set corresponding to all motifs of the condition were used. The motifs to be tested for each predicted autoantigen were selected using the following logical sequence: predicted autoantigen → microbial protein with matching epitope sequence → infectious organism → organism’s related autoimmune diseases → BED motifs.

The distribution of S for the predicted autoantigens was compared to the distribution for a random subset of 1000 samples from HPDS, as surrogate for a random distribution, using the one-sided Mann-Whitney *U* test (Di Bucchianicco, 1999).

#### 2.4.4 Prediction sets 5 and 6: SARS-CoV-2 peptide mimicry

Prediction sets 5 and 6 illustrate an actual application of the tool: the identification of potential human autoantigens resulting from SARS-CoV-2 peptide mimicry.

The analysis included those HLA class II alleles for which there is experimental evidence of their binding of human autoepitopes from HADS or SARS-CoV-2 epitopes from MEDS, plus a set of alleles from HLA-Spread (Dholakia et al., 2022) associated to autoimmune diseases that have been linked to SARS-CoV-2. As further filter, only those alleles for which there was a model in either CNN-PepPred or NetMHCIIpan were considered, leading to a total of 45 alleles (see Supplementary Material Table 3 file Alleles.xlsx).

We applied the same systems biology approach used on prediction sets 3 and 4 to explore potential functional relationships between predicted autoantigens and pathopysiological pathways associated to specific autoimmune diseases. To that end, we choose the BED motifs corresponding to the following autoimmune diseases associated with SARS-CoV-2 infection (see Supplementary Material Table 1 file MEDS_summary.xlsx): Anemia, Diabetes type 1, Guillain-Barre Syndrome, Myasthenia Gravis, Rheumatoyd Arthritis and, Lupus Erythematosus Systemic (Ehrenfeld et al., 2020; Knight et al., 2021). For each condition motif, the background distribution of the score S was calculated using proteins that had S>0 , lacked a predicted autoepitope, and were not present in the motif’s description. For the predicted autoantigens, the score S and its corresponding percentile, Perc (S) (indicating where it falls within the background distribution), were determined. Perc (S) served as an indicator of a potential functional relationship between the autoantigen and the pathophysiological motif. Thresholds were established as follows: Perc (S) > 95 indicated a weak functional relationship, Perc (S) > 99 indicated a functional relationship, and Perc (S) > 99.9 indicated a strong functional relationship.

## 3 Results and discussion

### 3.1 Supervised evaluation

Prediction sets 1 (using BLASTp) and 2 (using PSI-BLAST) evaluate the capacity of the tool to identify human peptides from a pool of known autoepitopes (HADS dataset) that significantly align with known epitopes from microbial species known to be associated to autoimmune diseases (MEDS dataset) and are recognised as HLA class II epitopes by one or both predictors used. In essence, starting from a pool of known human autoepitopes, the tool predicts which of them could induce autoimmunity as a consequence of a previous infection and microbial peptide mimicry. The raw results are reported in Supplementary Material Table 4 file MEDS_vs._HADS.xlsx.

Out of the 807 known human autoepitops contained in HADS, 21 had at least one sequence fragment that significantly aligned with a microbial epitope from MEDS and was predicted as an autoepitope by CNN-PepPred and/or NetMHCIIpan (Table 1). The matching microbial epitopes are from SARS-CoV-2, *Mycobacterium tuberculosis* (MT) and Human Alphaherpesvirus (HHV) 1 and 3. PSI-BLAST and BLASTp produced significant alignments in all cases.

**TABLE 1:** Results of the supervised evaluation.

Human autoepitope	AID	Microbial epitope	Organism	Same allele
H1-1 (62-70)	MS	PPE68 (63-68)	MT	-
H1-2 (59-67)	MS	PPE69 (63-68)	MT	-
H1-2 (203-211)	MS	HbhA (167-185)	MT	DRB5*01:01
H1-4 (210-218)	MS	HbhA (168-185)	MT	DRB5*01:01
H1-4 (210-218)	MS	RplV (152-157)	MT	DRB5*01:01
H2BC3 (66-74)	MS	EsxB (18-24)	MT	-
MPO (234-242)	MS	Spike (326-330)	SARS-CoV-2	DRB1*15:01
IFT57 (60-68)	MS	Spike (457-462)	SARS-CoV-2	-
PTPRJ (693-701)	MS	Spike (778-785)	SARS-CoV-2	-
RPL31 (103-116)	MS	Spike (1065-1069)	SARS-CoV-2	-
RPL7A (60-68)	MS	Spike (1210-1218)	SARS-CoV-2	-
ARID4B (540-552)	MS	Replicase polyprotein 1a (972-977)	SARS-CoV-2	-
CCDC97 (248-256)	MS	Replicase polyprotein 1a (972-977)	SARS-CoV-2	-
GLT8D1 (48-56)	MS	Replicase polyprotein 1a (1910-1917)	SARS-CoV-2	-
TGFBI (235-244)	MS	Replicase polyprotein 1a (2147-2155)	SARS-CoV-2	-
VIM (52-64)	MS	Replicase polyprotein 1a (3381-3389)	SARS-CoV-2	-
HLA-A (57-65)	MS	Replicase polyprotein 1a (3991-3999)	SARS-CoV-2	-
HLA-A (57-66)	RA	Replicase polyprotein 1a (3991-3999)	SARS-CoV-2	-
MRPS15 (196-204)	MS	Nucleoprotein (86-93)	SARS-CoV-2	-
PLXDC2 (38-49)	MS	Nucleoprotein (267-274)	SARS-CoV-2	-
ACTA2 (153-161)	MS	ORF3a protein (164-176)	SARS-CoV-2	-
H3-4 (21-29)	MS	ORF8 protein (51-56)	SARS-CoV-2	-
RPL5 (18-26)	MS	Tegument protein UL46 (486-490)	HHV-1	-
JAK2 (102-110)	MS	gE (38-42)	HHV-3	-

Human autoepitope: human protein name and position in the sequence of the predicted autoepitope; AID: autoimmune disease associated to the microorganism (MS: multiple sclerosis, RA: rheumatoid arthritis); Microbial epitope: microbial protein name and position in the sequence of the known microbial epitope; Organism: corresponding microrganism (MT: *Mycobacterium tuberculosis*, HHV: Human Alphaherpesvirus); Same allele: predicted allele, only shown when the same allele is known to recognise both human and microbial epitopes at the experimental level. An extended version of this table is provided in the Supplementary Material file MEDS_vs_HADS.xlsx.

In four of the cases, the predicted autoepitopes were matched at the epitope-allele level and using OneHLA as allele selection criteria (see Section 2.4.1), meaning that the predictions matched the allele that has been found to bind, experimentally, both the microbial and human epitopes. The corresponding autoantigens are H1-2, H1-4 and MPO, showing sequence similarity with HbhA and RplV from MT and the Spike Glicoprotein from SARS-CoV-2 (Table 1). Furthermore, the alleles DRB $1^{*}$ 15:01 and DRB $5^{*}$ 01:01 are known to be linked to the autoimmune disease—multiple sclerosis–that has been associated with these autoantigens (Karni et al., 1999; Finn et al., 2004; Prat et al., 2005; Z̆ivković et al., 2009; Shahbazi et al., 2010; Alcina et al., 2012; Quandt et al., 2012; Apperson et al., 2013; Kaushansky and Ben-Nun, 2014; Stürner et al., 2019).

### 3.2 Functional evaluation

Prediction sets 3 (using BLASTp) and 4 (using PSI-BLAST) evaluate the capacity of the tool to identify peptides from the full human proteome (HPDS dataset) that significantly align with known epitopes from microbial species known to be associated to autoimmune diseases (MEDS dataset) and are recognised as HLA class II epitopes by one or both predictors used. In short, it extends the analysis reported in Section 3.1 to the full human proteome. The raw results are reported in Supplementary Material Table 5 file MEDS_vs_HPDS.xlsx.

**FIGURE 2:**
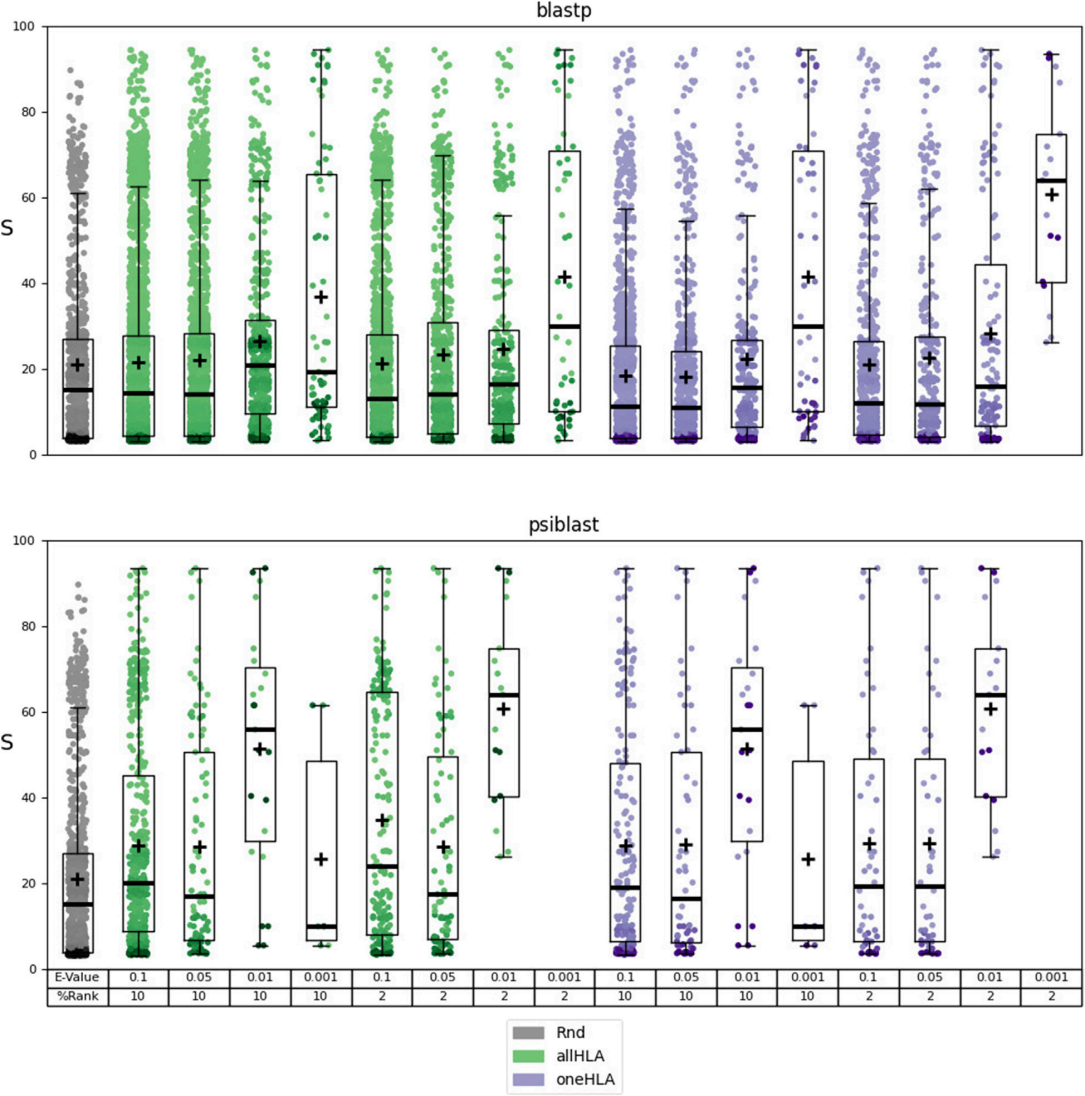
Results of the functional analysis for prediction sets 3 (using BLASTp) and 4 (using PSI-BLAST). Dependence of the distribution of scores S (box plots overlaid with scatterplots) on the following parameters: use of BLASTp or PSI-BLAST and threshold E-value for the alignment, %Rank threshold and allele-selection criterion (allHLA or one HLA, see Section 2.4.1) for the epitope prediction. The random distribution (Rnd) is represented in grey. The mean of the distribution is indicated with a cross.

The identified autoantigens were then subjected to the functional evaluation described in Section 2.4.3. The results of this evaluation are reported in Supplementary Material Table 6 file MEDS_functional.xlsx. The results are summarized in Figure 2 and Table 2, where the dependence of the distribution of scores S —the probability that there exists a relationship between the predicted autoantigen and a pathophysiological pathway associated to one of the autoimmune diseases considered–on the various parameters—E-value and %Rank thresholds, use of BLASTp or PSI-BLAST, use of the allHLA or oneHLA allele-selection criteria–is evaluated. In addition, the different score distributions are compared to a score distribution for a random subset of 1000 proteins from HPDS.

**TABLE 2:** Results of the functional analysis for prediction sets 3 (using BLASTp) and 4 (using PSI-BLAST).

Alignment	Alleles	%Rank	E-Value	N	Score ratio	*p*-value	Significance
BLASTp	allHLA	10	0.1	3144	1.11	3.41e-02	-
BLASTp	allHLA	10	0.05	1770	1.15	2.33e-02	-
BLASTp	allHLA	10	0.01	516	1.36	1.10e-13	***
BLASTp	allHLA	10	0.001	67	1.91	2.05e-06	***
BLASTp	allHLA	2	0.1	1594	1.1	1.99e-01	-
BLASTp	allHLA	2	0.05	794	1.2	1.07e-02	-
BLASTp	allHLA	2	0.01	301	1.28	4.25e-04	**
BLASTp	allHLA	2	0.001	50	2.14	9.51e-07	***
BLASTp	oneHLA	10	0.1	1346	0.96	9.88e-01	-
BLASTp	oneHLA	10	0.05	711	0.95	9.82e-01	-
BLASTp	oneHLA	10	0.01	299	1.15	1.26e-02	-
BLASTp	oneHLA	10	0.001	50	2.14	9.51e-07	***
BLASTp	oneHLA	2	0.1	618	1.09	3.53e-01	-
BLASTp	oneHLA	2	0.05	330	1.16	2.95e-01	-
BLASTp	oneHLA	2	0.01	134	1.47	2.43e-03	*
BLASTp	oneHLA	2	0.001	17	3.14	9.69e-10	***
PSI-BLAST	allHLA	10	0.1	487	1.49	1.53e-13	***
PSI-BLAST	allHLA	10	0.05	89	1.47	6.60e-04	**
PSI-BLAST	allHLA	10	0.01	23	2.66	3.85e-08	***
PSI-BLAST	allHLA	10	0.001	6	1.33	3.26e-01	-
PSI-BLAST	allHLA	2	0.1	184	1.8	3.03e-11	***
PSI-BLAST	allHLA	2	0.05	83	1.48	6.45e-04	**
PSI-BLAST	allHLA	2	0.01	17	3.14	9.69e-10	***
PSI-BLAST	allHLA	2	0.001	0	-	-	-
PSI-BLAST	oneHLA	10	0.1	188	1.49	1.22e-05	***
PSI-BLAST	oneHLA	10	0.05	56	1.5	1.04e-02	-
PSI-BLAST	oneHLA	10	0.01	23	2.66	3.85e-08	***
PSI-BLAST	oneHLA	10	0.001	6	1.33	3.26e-01	-
PSI-BLAST	oneHLA	2	0.1	50	1.52	1.08e-02	-
PSI-BLAST	oneHLA	2	0.05	50	1.52	1.08e-02	-
PSI-BLAST	oneHLA	2	0.01	17	3.14	9.69e-10	***
PSI-BLAST	oneHLA	2	0.001	0	-	-	-

Alleles: allele-selection criterion (see Section 2.4.1); Score ratio: mean-score ratio; N: size of the sample; *p*-value: from the Mann-Whitney *U* test; Significance: - (not significant), * (*p*-value < 0.01), ** (*p*-value < 0.001), *** (*p*-value < 0.0001).

Using PSI-BLAST notably reduces the number of hits, but its strength lies in incorporating specific biological information into the alignments. By leveraging the pool of peptides known to bind HLA class II molecules to generate a Position-Specific Scoring Matrix, PSI-BLAST may enhance the alignment’s relevance. Although this approach results in fewer hits, it achieves the highest mean score ratio and the most significant distinction between predicted autoantigen score distributions and the random peptide score distribution, particularly at E-value and %Rank thresholds of 0.01 and 2, respectively. These thresholds optimize the selection of predicted autoantigens, increasing the likelihood of identifying proteins associated with a specific autoimmune disease in the human protein network. However, using a more stringent E-value threshold of 0.001 drastically reduces the number of aligned sequences, which, when combined with a %Rank threshold of 2, can prevent the prediction of any autoantigens. The choice of allele-selection criteria for the binding prediction, allHLA or oneHLA, plays also a significant role in the results, particularly regarding the number of hits.

The results of this analysis require some biological context for proper interpretation. We are examining the potential relationship, within a graph representing the human protein network, between a protein identified as a potential autoantigen and a set of proteins that have been linked to the pathophysiology of a specific autoimmune disease. Clearly, the proteins in this set tend to be elements of the immune system. Therefore, a high score indicates that the predicted autoantigen is, in network terms, associated to these immune-system elements. While this may not be a universal characteristic of all autoantigens, Figure 2 and Table 2 show that as we apply more stringent alignment and epitope prediction thresholds, thereby increasing our confidence in the autoepitope, the mean score and significance of the relationship between autoantigen and autoimmune disease motif also increase. This focused approach not only enhances the reliability of our predictions but also allows us to propose potential links between the predicted autoantigens and specific autoimmune diseases, which would be difficult to establish otherwise.

### 3.3 SARS-CoV-2 peptide mimicry

#### 3.3.1 The predicted autoantigens and their relation to autoimmune diseases

Prediction sets 5 (using BLASTp) and 6 (using PSI-BLAST) evaluate the capacity of the tool to identify peptides from the full human proteome (HPDS dataset) that significantly align with known epitopes from SARS-CoV-2 (SC2DS dataset) and are recognised as HLA class II epitopes by one or both predictors used. It thus focuses the analysis on finding SARS-CoV-2 epitopes that could induce an autoimmune disease through peptide mimicry. The raw results are reported in Supplementary Material Table 7 file SC2DS_vs_HPDS.xlsx. As in the previous section, the identified autoantigens were evaluated for their potential relationship with autoimmune-disease motifs in the human protein network (Section 2.4.3). The results of this evaluation are reported in Supplementary Material Table 8 file SC2DS_functional.xlsx.

Using BLASTp, MMPred identified 14 potential autoantigens: MYT1L, BAZ1A, CHD5, MCM8, ATF7, MOV10, MOV10L1, DNA2, BRI3, PARVG, CALD1, MICAL3, SLC35E4, and UNC50, using a threshold E-value of 0.01. In contrast, PSI-BLAST did not detect any autoepitopes with an E-value below 0.01 and predicted only one autoepitope from HELZ2 with an E-value below 0.05. This outcome is consistent with the previous section’s analysis, where BLASTp yielded a significantly higher number of positive predictions at the same thresholds. Most alignments involved SARS-CoV-2 Non-Structural Proteins (NSPs), particularly NSP3, NSP5, NSP13, NSP14, NSP15, and NSP16. Additionally, one alignment involved Nucleoprotein N, and two alignments were related to the Spike protein of the Omicron variants BA.1-like and BA.4-like (Wu et al., 2020). These findings are summarized in Table 3.

**TABLE 3:** MMPred-predicted autoantigens from similarity to SARS-CoV-2 sequences.

Autoantigen	Epitope	SARS-CoV-2 protein	Aligned sequence	N positive
MYT1L	EEGDREEEE (125–133)	NSP3	DEDEEEGDCEEEE (110–122)	1
BAZ1A	VDGDEEEGQSEEEE (1229–1242)	NSP3	DEDEEEGDCEEEEFE (110–124)	2
CHD5	DDDDEEEEGGCEEEED (398–413)	NSP3	DEDEEEGDCEEEE (110–122)	2
MCM8	YNYEPLTQL (199–207)	NSP5	YNYEPLTQ (237–244)	1
ATF7	FVCNAPGCG (7–15)	NSP13	PYVCNAPGC (47–55)	1
HELZ2	FTVIQGPPG (2169–2177)	NSP13	QKYSTLQGPPGTGKS (275–289)	11
MOV10	KRFNVAVTRAKAL (903–915)	NSP13	NVNRFNVAITRAK (557–569)	5
MOV10L1	RFNVAITRPKAL (1131–1142)	NSP13	NVNRFNVAITRAK (557–569)	5
DNA2	LNVAITRAKH (1000–1009)	NSP13	RFNVAITRAK (560–569)	4
BRI3	VTRYPANSI (64–72)	NSP14	VDRYPANSIV (389–398)	7
PARVG	LHLLVALAKRFQ (140–151)	NSP15	LHLLIGLAKRF (248–258)	8
CALD1	VMSLKNGQI (225–233)	NSP16	TAVMSLKEGQI (257–267)	2
MICAL3	YKKDKKKKA (1747–1755)	N	KKDKKKKADE (369–378)	3
SLC35E4	SVLYNLASF (265–273)	spike(BA.1-like)	SVLYNLASFS (366–375)	1
UNC50	YKYLRRLFR (32–40)	spike(BA.4-like)	YNYLRRLFR (447–455)	7

N positive: number of positive predictions for the epitope (pairs of prediction method and allele).

**FIGURE 3:**
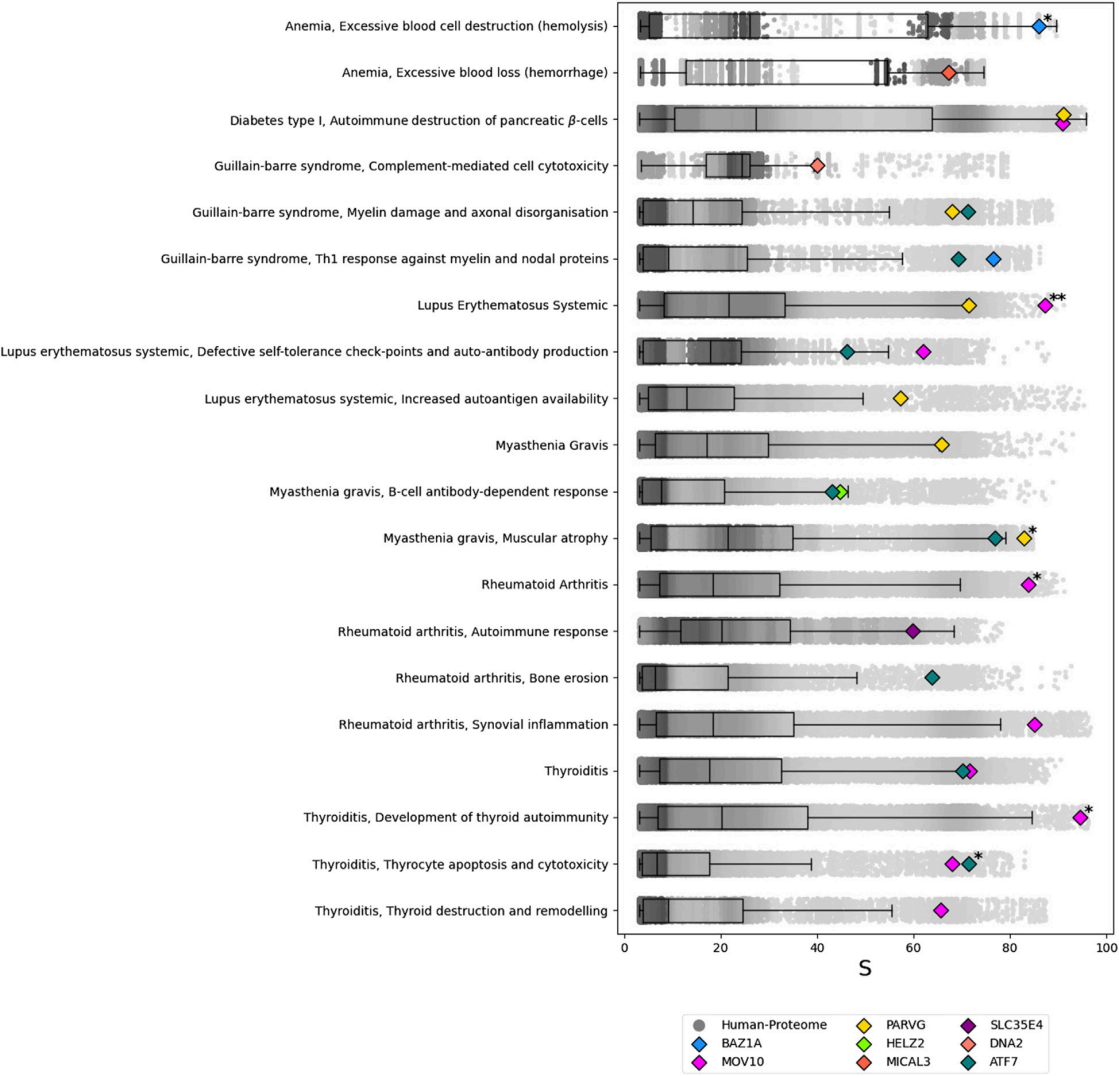
Results of the ANNs analysis for the predicted autoantigens of the SARS-CoV-2 vs. Human protome prediction sets. For each of the autoimmune-disease motifs tested, a boxplot with overlapped scatterplot represent the background distribution of the score S . The eight predicted autoantigens that satisfy Perc(S) > 95 are shown. Those with Perc(S) > 99 are marked with * and those with Perc(S) > 99.9 are marked with **.

In the functional analysis, the 15 predicted autoantigens were assessed for potential associations with 32 autoimmune-disease motifs annotated in the network. Eight proteins—BAZ1A, ATF7, MOV10, DNA2, PARVG, MICAL3, SLC35E4, and HELZ2– scored in the 95th percentile (Perc (S) , see Section 2.4.4) or higher for 19 different motifs (see Figure 3). While no autoantigen met the minimum threshold for certain motifs, at least one motif from each selected autoimmune disease had a significant hit. Notably, BAZ1A, MOV10, and PARVG were the only proteins with a Perc (S) exceeding 99 for at least one motif. The highest Perc (S) (above 99.9) was achieved by MOV10 in association with the “Lupus Erythematosus Systemic” motif.

With a Perc (S) > 95, Rheumatoid Arthritis and Thyroiditis are associated with the same predicted autoantigens, MOV10 and ATF7, which are also linked to Lupus Erythematosus Systemic along with PARVG. Additionally, SLC35E4 is also connected to Rheumatoid Arthritis. Type I Diabetes is associated with the predicted autoantigens MOV10 and PARVG, with PARVG also linked to Guillain-Barré Syndrome, alongside ATF7, BAZ1A, and DNA2. Myasthenia Gravis shows predicted associations with HELZ2, ATF7 and PARVG. Lastly, Anemia is associated with MICAL3 and BAZ1A.

When the threshold is raised to Perc (S) > 99, MOV10 is associated with both Lupus Erythematosus Systemic and Rheumatoid Arthritis. MOV10 is also linked to Thyroiditis, along with ATF7. Anemia is connected to BAZ1A, and Myasthenia Gravis is linked to PARVG.

The human proteins MYT1L, CHD5, MCM8, MOV10L1, BRI3 and CALD1 were predicted as autoantigens but did not show a Perc (S) > 95 for any of the motifs tested.

#### 3.3.2 The predicted human autoepitopes align with known SARS-CoV-2 epitopes

While there is no experimental evidence in IEDB linking our predicted autoepitopes to autoimmune diseases, many of the SARS-CoV-2 sequences that align significantly with these predicted autoepitopes are known to bind HLA class II molecules. Specifically, the SARS-CoV-2 sequences 369-378 of the Nucleoprotein, 47-55, 275-289, and 557-569 of NSP13, 389-398 of NSP14, 248-258 of NSP15, and 257-267 of NSP16 overlap with regions that have been experimentally validated (Obermair et al., 2022; Huisman et al., 2022) (see Table 3). Notably, the regions 275-289 and 557-569 of NSP13 show allele-specific hits, where the autoepitopes have been predicted for the same alleles experimentally observed to bind these SARS-CoV-2 protein regions. Thus, DRB $1^{*}$ 07:01 is predicted to bind an autoepitope in MOV10, and DRB $1^{*}$ 04:04 is predicted to bind an autoepitope in HELZ2.

The functional analysis reveals an interesting pattern among the predicted autoantigens that align with known SARS-CoV-2 epitopes. Specifically, nine human proteins—ATF7, HELZ2, MOV10, MOV10L1, DNA2, BRI3, PARVG, CALD1, MICAL3– exhibit significant alignment with these SARS-CoV-2 epitopes. Among these, ATF7, HELZ2, MOV10, DNA2, PARVG and MICAL3 rank in the 95th percentile or higher (Perc (S) > 95) across 18 different motifs, with ATF7, MOV10, and PARVG showing Perc (S) > 99 for six motifs. MOV10 alone achieves a Perc (S) > 99.9 for a single motif. In contrast, the associations are less significant when examining predicted autoantigens derived from alignments with SARS-CoV-2 sequences not known to be antigenic. Of the six proteins with peptides matching these regions (MYT1L, BAZ1A, CHD5, MCM8, SLC35E4 and UNC50), only BAZ1A and SLC35E4 yield significant results, both achieving Perc (S) > 95 for just three motifs, with only BAZ1A reaching Perc (S) > 99 for a single motif.

#### 3.3.3 The antiviral activity of the predicted autoantigens MOV10 and HELZ2

We examined baseline expression patterns of the predicted autoantigens in lung cells using the Expression Atlas (George et al., 2024). Proteomics data from Wang et al. (2019) indicate that 11 out of the 15 predicted autoantigens (BAZ1A, MCM8, ATF7, HELZ2, MOV10, MOV10L1, DNA2, PARVG, CALD1, MICAL3, UNC50) are highly expressed, supporting the hypothesis that cross-reaction with HLA class II molecules in previously infected lung cells is plausible (Kawasaki et al., 2022; Hoffmann et al., 2020). Additionally, a gene enrichment analysis conducted using the online tool g:Profiler (Kolberg et al., 2023) (threshold = 0.05, multiple hypothesis testing method g:SCS) revealed significant enrichment for the Gene Ontology (GO, https://geneontology.org/) Molecular Function terms “helicase activity” (GO:0004386) and “single-stranded DNA-helicase” (GO:0017116), as well as the Cellular Component terms “P granule” (GO:0043186) and “intracellular non-membrane-bounded organelle” (GO:0043232). Notably, the human proteins MOV10, HELZ2, and DNA2, which are reported to have helicase activity with GO evidence code “inferred by direct assay” (IDA), contain segments that align with the helicase NSP13 of SARS-CoV-2 (see Table 3). MOV10 is implicated in the modulation of viral infectivity (Goodier et al., 2012) and promotes type I interferon production (Cuevas et al., 2016; Yang et al., 2022; Balinsky et al., 2017), while HELZ2 is known to respond to interferon production during viral infection (Huntzinger et al., 2023; Du et al., 2024). DNA2 does not appear to have any known antiviral activity. On the other hand, the helicase activity of NSP13 is crucial for viral replication (Yan et al., 2021) and this protein interacts with the host to inhibit interferon-beta production, thereby evading the immune response (Xia et al., 2020). Thus, it seems that MOV10 and HELZ2 play roles antagonistic to that of NSP13.

Overall, transcriptomic and proteomic data on our predicted autoantigens in the context of SARS-CoV-2 infection primarily focus on MOV10 and HELZ2. These studies utilize samples obtained from either the lungs of infected patients or lung cell lines.

Wang et al. employed Dermatan Sulfate (DS)-affinity proteomics to define the autoantigen-ome of lung fibroblasts, complemented by bioinformatics analyses to explore the relationship between autoantigenic proteins and COVID-19-induced alterations (Wang et al., 2022). Notably, they discovered that 86% of their predicted autoantigens were either up- or downregulated in COVID-19 patients or SARS-CoV-2-infected cells. Among the previously unknown autoantigens identified in this study, MOV10 (with very high DS affinity) and CALD1 (with medium to high DS affinity) align with our predictions. Both proteins exhibited altered expression in COVID-19 patients and/or SARS-CoV-2-infected cells.

In a study by An et al., the authors developed a bioinformatics pipeline similar to ours (as noted in the Introduction) and conducted a differential expression analysis using the Calu-3 human lung adenocarcinoma cell line (An et al., 2022). Their pipeline predicts that MOV10 and HELZ2 may exhibit cross-reactivity, while expression data reveal a positive correlation between transcript abundance of these proteins, particularly HELZ2, and SARS-CoV-2 viral load in infected cells.

A study by Ariumi explored the epigenetic mechanisms triggered by SARS-CoV-2 infections (Ariumi, 2022). The findings indicate that the knockdown of MOV10 leads to a significant increase in viral load/replication in infected cells, suggesting that MOV10 plays a role in the host’s suppression of SARS-CoV-2 replication.

A study examining gene expression in cell lines and patient samples in the context of epigenetic regulation during SARS-CoV-2, SARS-CoV, and MERS infections identified various differentially expressed genes involved in the epigenetic response during infection in pulmonary cell lines (Salgado-Albarrán et al., 2021). Although the study does not report expression data for MOV10, it highlights MOV10’s functional and physical relationships with the differentially expressed genes through protein-protein interaction (PPI) data (Kotlyar et al., 2019) and co-expression analysis (Langfelder and Horvath, 2008).

Aside from MOV10 and HELZ2, the literature also reports the overexpression of MICAL3 in convalescent COVID-19 patients who retested positive (Fang et al., 2022). However, no relevant studies have been found that link the other predicted autoantigens to SARS-CoV-2.

#### 3.3.4 Sequence and structural similarity between NSP13 and MOV10, an immune-excape mechanism?

Considering the reported evidence regarding MOV10, we extended our investigation to NSP13 due to its shared helicase activity and the presence of a similar HLA class II epitope. Given MOV10’s suggested suppressor role in SARS-CoV-2 replication, its presence in lung epithelial cells, its upregulated expression in SARS-CoV-2-infected cells, and the APC-like properties of lung epithelial cells (Kawasaki et al., 2022; Hoffmann et al., 2020), we propose that cross-reactive epitopes in NSP13 could potentially trigger autoimmunity and facilitate SARS-CoV-2 replication in the lungs.

We would therefore expect the NSP13 epitope NVNRFNVAITRAK (positions 557 to 569, see Table 3) to be conserved across NSP13 variants. However, it is important to note that this conservation might also arise from the sequence’s involvement in NSP13’s catalytic activity (Newman et al., 2021).

**FIGURE 4:**
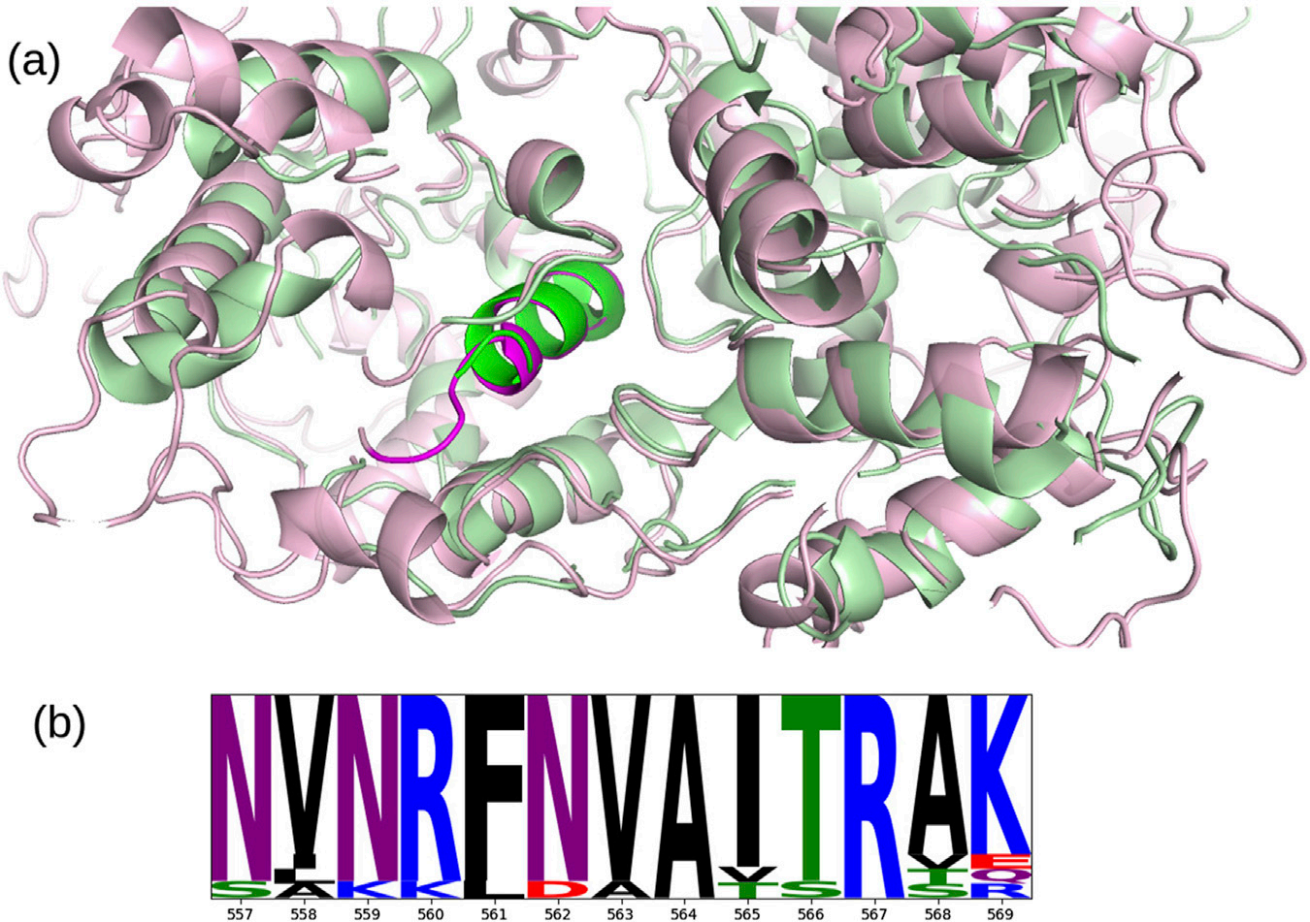
**(A)** Superposition of the crystallographic structure of NSP13 (PDB entry 6ZSL, chain B) (green) with the predicted AlphaFold structure of MOV10 (UniProt entry Q9HCE1) (pink), the highlighted α -helices in the center correspond to residues 557-569 of NSP13 and 901-913 of MOV10. Superposition and image were generated with PyMOL (https://www.pymol.org/). **(B)** Logo plot of the multiple sequence alignment for the NSP13 epitope. To facilitate visualization, a pseudocount of 0.1 is used and a min-max normalization of each position is applied. Image generated with the logomaker python package (Tareen and Kinney, 2020).

A total of 1,725,419 protein sequences of the ORF1ab polyproteins were downloaded from NCBI Virus (15/02/2024) (Hatcher et al., 2017), with the maximum number of ambiguous characters set to zero, and considering only sequences collected for baseline surveillance (random sampling = “Only”). To extract NSP13 from the ORF1ab sequences, a BLASTp alignment was performed using the reference sequence of NSP13 as the query and the ORF1ab sequences as the target. Due to the high similarity of the variants to the reference, all alignments yielded significant results, allowing us to heuristically extract the corresponding NSP13 variants. A multiple sequence alignment of all NSP13 variants, along with the NSP13 reference, was then performed using FAMSA (Deorowicz et al., 2016), which is optimized for datasets with high dimensionality and high pairwise identity. To compute the conservation of the NSP13 epitope, we averaged the Shannon Entropy (Shenkin et al., 1991) across its positions and compared it to the distribution of all windows of the same length across the alignment. The NSP13 epitope at positions 557 to 569 showed a higher conservation score than 95.7% of the windows of the same size, indicating the strong conservation of this region. A logo plot of the alignment is presented in Figure 4.

Furthermore, the superposition of the crystallographic structure of NSP13 (PDB entry 6ZSL, chain B) (Newman et al., 2021) with the predicted AlphaFold structure of MOV10 (UniProt entry Q9HCE1) (Jumper et al., 2021; Varadi et al., 2022) reveales significant structural similarity between two regions of these proteins of 214 residues in length (36.5% of the sequence of 6ZSL and 21.3% of that of the AlphaFold model of Q9HCE1, with a ${\text{C}}_{\alpha }$ RMSD of 1.98 Å, see Figure 4). Notably, the regions corresponding to the epitopes—NSP13 residues 557-569 and MOV10 residues 901-913– are superimposed in the structural alignment, suggesting that epitope mimicry could arise from both sequence and structure. Although the MOV10 structure is based on an AlphaFold model (Terwilliger et al., 2024), the model confidence in this region is very high (pLDDT > 90).

The molecular mimicry mechanism hypothetically employed by SARS-CoV-2 may parallel other viral strategies identified as triggers for autoimmune diseases (Zhao et al., 1998; Hrycek et al., 2005; Bjornevik et al., 2022), functioning in two potential ways as outlined by Maguire et al. (2024): *i)* by mimicking a structural region of MOV10, NSP13 could enable SARS-CoV-2 to evade immune detection and achieve immune tolerance, facilitating viral persistence and diminishing activation of the host’s adaptive immune response (Maguire et al., 2024; Cusick et al., 2011; Zhao et al., 1998; Adiguzel, 2021); *ii)* NSP13’s sequence and structural resemblance to MOV10 might competitively inhibit MOV10’s activity, impairing its antiviral function in lung epithelial cells and promoting SARS-CoV-2 replication. Additionally, the similarity between NSP13 and MOV10 may induce the development of cross-reactive T-cells and/or antibodies via HLA class II epitope recognition, potentially triggering an autoimmune response (Cusick et al., 2011; Smatti et al., 2019; Zhao et al., 1998; Sabbatini et al., 1993).

It is noteworthy that these mechanisms could operate concurrently, with NSP13’s epitope conservation enhancing SARS-CoV-2 infection in both scenarios. The predominance of one mechanism over the other likely depends on the specific HLA class II alleles present, as different alleles are associated with distinct outcomes (Dholakia et al., 2022).

Overall, these findings support the hypothesis of cross-reactivity between the epitopes of NSP13 and MOV10, which may influence SARS-CoV-2 replication in the lungs. While the potential for an autoimmune response against MOV10 and its connection to autoimmune diseases remains speculative at this stage (see Section 3.3.1), further investigation is warranted.

## Data Availability

The original contributions presented in the study are included in the article/Supplementary Material, further inquiries can be directed to the corresponding author.
